# The Influence of Preparation Temperature on the Different Facets of Bulk MgB_2_ Superconductors

**DOI:** 10.3390/mi14050988

**Published:** 2023-04-30

**Authors:** Penghe Zhang, Yufeng Zhang, Chunyan Li, Yan Zhang, Shuangyuan Shen, Guanjie Ruan, Jiaying Zhang, Jacques Guillaume Noudem

**Affiliations:** 1 College of Mathematics and Physics, Shanghai University of Electric Power, Shanghai 201306, China; 2 Shanghai Key Laboratory of High Temperature Superconductors, Shanghai University, Shanghai 200444, China; 3 Normandie University, ENSICAEN, UNICAEN, CNRS, CRISMAT, 14000 Caen, France

**Keywords:** MgB_2_ bulk superconductor, spark plasma sintering, critical temperature, critical current density, pinning mechanism

## Abstract

Two MgB_2_ samples were prepared using the spark plasma sintering (SPS) technique at different temperatures—950 °C (S1) and 975 °C (S2)—for 2 h under 50 MPa pressure to study the influence of preparation temperature on different facets, namely those perpendicular (PeF) and parallel (PaF) to the compression direction of uniaxial pressure during the SPS of MgB_2_ samples. We analyzed the superconducting properties of the PeF and PaF of two MgB_2_ samples prepared at different temperatures from the curves of the critical temperature (*T*_C_), the curves of critical current density (*J*_C_), the microstructures of MgB_2_ samples, and the crystal size from SEM. The values of the onset of the critical transition temperature, *T*_c,onset_, were around 37.5 K and the transition widths were about 1 K, which indicates that the two samples exhibit good crystallinity and homogeneity. The PeF of the SPSed samples exhibited slightly higher *J*_C_ compared with that of the PaF of the SPSed samples over the whole magnetic field. The values of the pinning force related to parameters *h*_0_ and *K*_n_ of the PeF were lower than those of the PaF, except for *K*_n_ of the PeF of S1, which means that the PeF has a stronger GBP than the PaF. In low field, the most outstanding performance was S1-PeF, whose critical current density (*J*_C_) was 503 kA/cm^2^ self-field at 10 K, and its crystal size was the smallest (0.24 µm) among all the tested samples, which is consistent with the theory that a smaller crystal size can improve the *J*_C_ of MgB_2_. However, in high field, S2-PeF had the highest *J*_C_ value, which is related to the pinning mechanism and can be explained by grain boundary pinning (GBP). With an increase in preparation temperature, S2 showed a slightly stronger anisotropy of properties. In addition, with an increase in temperature, point pinning becomes stronger to form effective pinning centers, leading to a higher *J*_C_.

## 1. Introduction

Since Akimitsu [1] discovered the superconductivity of MgB_2_, it had been studied extensively by scientists from all over the world [2], and researchers have processed MgB_2_ into tapes, wires [3], films [2,4], and bulks based on in situ and ex situ methods [5]. Compared to low-temperature superconductors, MgB_2_ has a higher critical temperature (*T*_C_) and a higher critical current density (*J*_C_) [6,7,8]. However, it has a lower coherence length, lower anisotropy, and a good grain boundary connection compared with high-temperature superconductors [9]. More important is that its raw materials, magnesium and boron, are abundant on Earth [10], which means that its preparation is much less expensive than that of REBa_2_Cu_3_O_7-*x*_ (RE represents a rare Earth element, such as Gd, Nd, Sm, etc.) bulk superconductors [11,12,13]. It is also speculated that, according to the raw materials of MgB_2_ superconducting bulks, there are no toxic elements in their ingredients, which is relatively safe and conducive to environmental protection [10]. All these characteristics make MgB_2_ a promising superconducting material for NMR/MRI magnets [14], motors/generators [15], etc. Most of these applications need a strong trapped magnetic field that is proportional to the critical current density, *J*_C_, and sample size according to the Bean model [16]. Therefore, when the sample size is determined, the improvement of *J*_C_ related to the effective pinning centers becomes the best way to obtain a stronger trapped magnetic field, and the preparation methods that can improve the *J*_C_ of MgB_2_ samples become research hotspots. Therefore, several studies about the preparation methods for MgB_2_ have been published, such as hot pressing [17], irradiation [18], doping/adding [19,20], and spark plasma sintering [6], which has not only caused the improvement of its preparation methods, but also a higher *J*_C_, which is linked to properties and practical applications [21,22,23]. As one of these methods, the spark plasma sintering (SPS) technique can greatly suppress grain-coarsening and prepare high-density samples [8,21] successfully in a short time, even in less than two hours. The superconductor bulk MgB_2_ prepared at a temperature of 850 °C following the ex situ method and using the SPS technique shows the best *J*_C_ in the whole field, compared with bulks prepared at different temperatures, namely 800 °C, 850 °C, 900 °C, and 1000 °C [5].

It is known that MgB_2_ is an anisotropic material [24,25], which means its properties may be different in different directions. The SPS process is carried out under uniaxial pressure, so there are perpendicular (PeF) and parallel (PaF) facets to the compression direction of the uniaxial pressure. The effects on the different facets under various sintering durations [7] at 950 °C have been reported. In this study, two kinds of MgB_2_ bulks are prepared at different temperatures—950 °C, marked by S1, and 975 °C marked by S2—under a uniaxial pressure of 50 MPa for 2 h using the SPS technique to analyze the properties of MgB_2_ bulk samples. The influence of different processed temperatures on different facets of SPSed MgB_2_ bulks is discussed from superconducting performance and microstructure points of view. Through the analysis of the flux-pinning mechanism, grain boundary pinning (GBP) and point pinning (PP) were discussed.

## 2. Experimental Section

Grade A magnesium diboride from ABCR GmbH (Karlsruhe, Germany) was used as the starting powder and then loaded into a graphite die and processed using the spark plasma sintering technique (FCT System GmbH, HD25, Rauenstein, Germany) in DC mode. A pulsed electric current (2000 A, 4 V) was passed through the sample under a dynamic vacuum (10^−3^ bar) while a 50-MPa uniaxial pressure was applied [22]. In this study, two SPSed bulk samples with disc shapes 20 mm in diameter and 3 mm in thickness were fabricated at different temperatures—950 °C (S1) and 975 °C (S2)—for 2 h under a pressure of 50 MPa.

**Figure 1:**
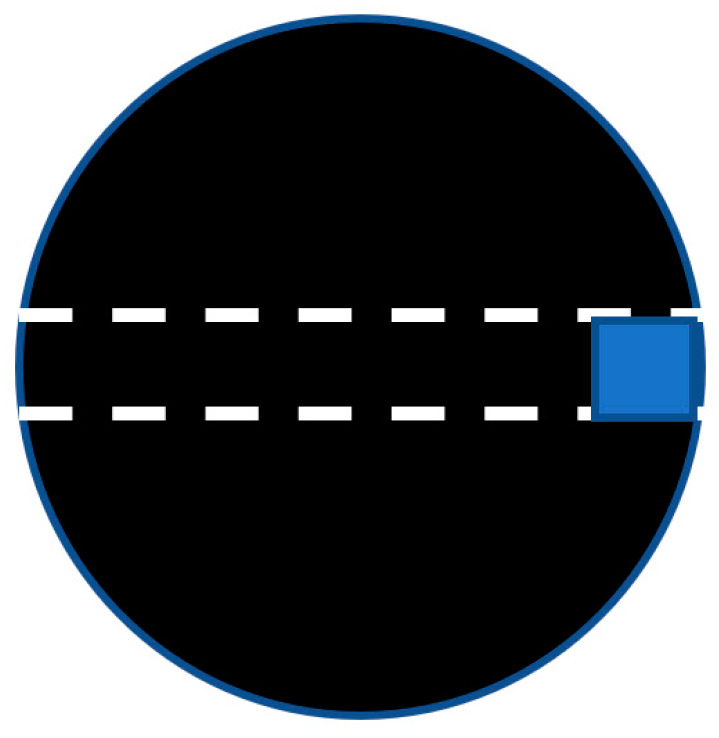
Schematic diagram of specimens cut in the position of both MgB_2_ bulks.

To study the effect of preparation temperature on the performance of MgB_2_ bulks, small specimens of rectangular shape positioned beneath the edge of MgB_2_ bulk samples were cut into sizes of 2 mm × 2 mm × 1 mm from these bulk samples for the measurement of superconducting properties, as shown in Figure 1. The phase position and lattice parameters were confirmed using X-ray diffraction (XRD) at room temperature. Scanning electron microscopy (SEM, microscope model, company, city, country)) was used to observe the microstructure of these samples, and the crystal size of the SPSed MgB_2_ was calculated according to the SEM using Nano Measurer 1.2. The element analysis was conducted using energy-dispersive X-ray spectroscopy (EDS). The DC magnetization measurement was measured using a Quantum Design SQUID magnetometer under a magnetic field perpendicular to the tested facet for the superconducting transition and magnetic hysteresis loops of MgB_2_ samples. The values of critical current density (*J*_C_) were calculated based on the extended Bean’s critical state model [16,26]. The facets perpendicular to the compression direction in the SPS process were marked as perpendicular facets (PeF), and the facets parallel to the compression direction were marked as parallel facets (PaF). The two kinds of facets were compared to study the differences in superconducting properties between the PeF and PaF.

## 3. Results

### 3.1. Superconducting Properties of the PeF and PaF of MgB_2_ Samples at Different Preparation Temperatures (950 °C and 975 °C)

**Figure 2:**
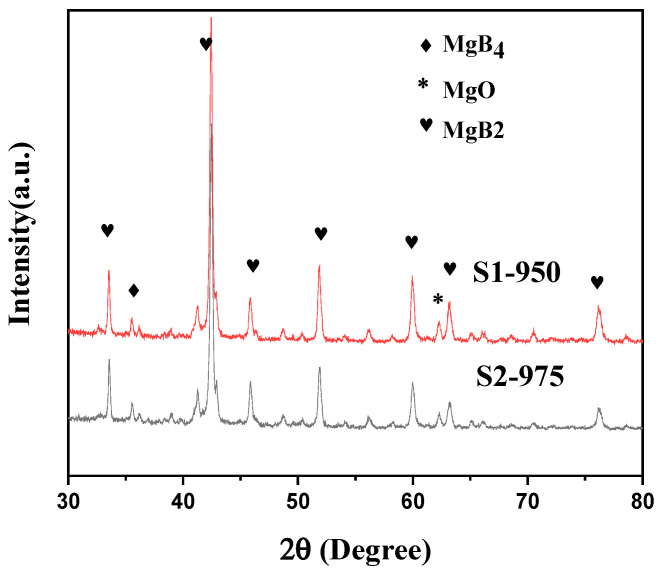
Room-temperature X-ray diffraction patterns of samples prepared using the spark plasma sintering method at 950 °C (S1) and 975 °C (S2) for 2 h under 50 MPa pressure.

Figure 2 shows the room-temperature XRD patterns of samples prepared at 50 MPa uniaxial pressure and different temperatures, 950 °C (S1) and 975 °C (S2), using spark plasma sintering technology. It can be seen from Figure 2 that the main component of the samples is MgB_2_, although there are a few second phases, such as MgB_4_ and MgO. The oxidation reaction appears under the oxygen condition during growth [21,27]. High temperature and pressure conditions during the process [27,28] may result in the generation of MgB_4_. These kinds of particles may have an important effect on the superconducting properties of SPSed samples.

The standard lattice parameter values of MgB_2_ without doping any other substances are a = 3.086 Å, c = 3.524 Å. Table 1 shows that the lattice parameters of the samples at different temperatures, 950 °C (S1) and 975 °C (S2), are a = 3.0847 Å, 3.0811 Å, c = 3.5232 Å, and 3.5223 Å, respectively. A small a-axis lattice parameter is reflected in the substitution of carbon from the graphite mold system for boron in the crystal lattice of MgB_2_ during the process [29,30]. The lattice parameters of S1 are closer to the standard values of MgB_2_. Therefore, we can infer that the performance of the S1 sample may be slightly better than that of the S2 sample.

**Figure 3:**
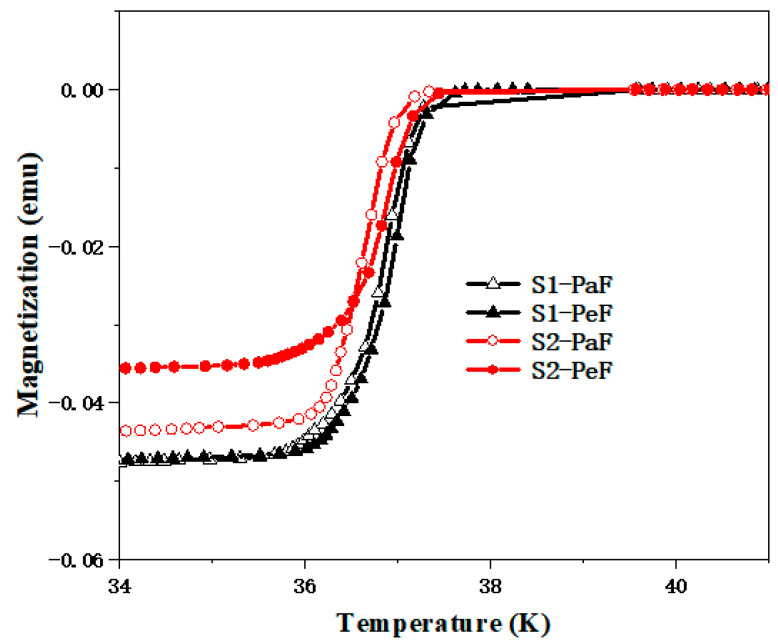
The *T*_C_ (temperature–magnetization) curves of the SPSed MgB_2_ bulk samples prepared at different temperatures of 950 °C (S1) and 975 °C (S2) for 2 h under 50 MPa pressure.

Figure 3 shows the sample temperature dependence of magnetization curves of MgB_2_ samples prepared at different temperatures of 950 °C (S1) and 975 °C (S2) for 2 h under 50 MPa pressure. The onset of the critical transition temperature, *T*_c,onset_, is at around 37.5 K and the transition width is about 1 K, which indicates the two samples exhibit good crystallinity and homogeneity. The *T*_c,onset_ of S1 is slightly higher than that of the S2 sample, which may be related to the good performance of the S1 sample.

**Figure 4:**
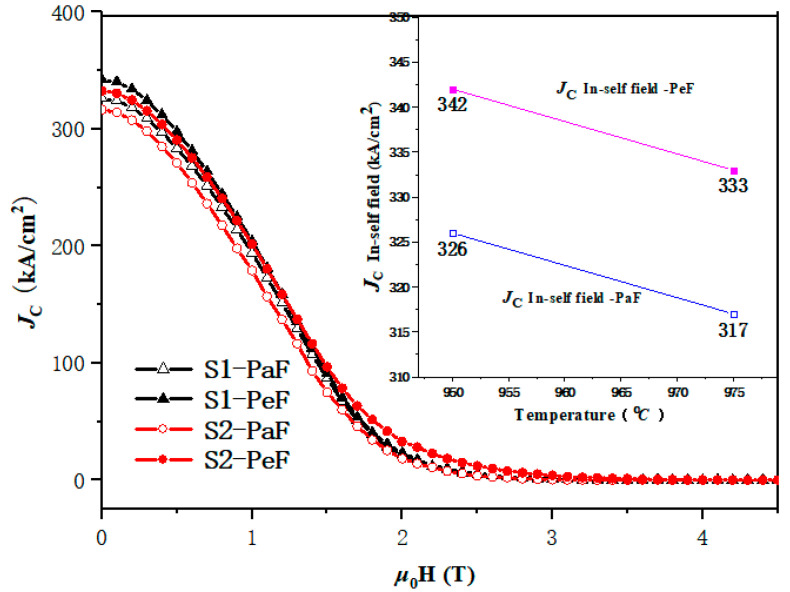
The *J_C_*-*μ*_0_H curves of magnetic field and critical current density at 20 K of two surfaces, PeF and PaF, of SPSed MgB_2_ bulk samples. The inset represents the critical current density measured at the self-field.

Figure 4 shows the curves of the magnetic field and *J*_C_ from different facets (PeF and PaF) measured at 20 K, and the embedded diagram shows their self-field *J*_C_ at different preparation temperatures. It can be seen that *J*_C_ decreases with the increase in the magnetic field. The PeF of the SPSed samples exhibits a slightly higher *J*_C_ compared with that of the PaF of the SPSed samples in the whole magnetic field. In the low magnetic field, the highest self-field *J*_C_ is 342 kA/cm^2^ in the S1-PeF samples, as shown in Figure 4. The self-field *J*_C_ of the S2-PeF sample is 333 kA/cm^2^. The self-field of *J*_C_ in the PaF of the S1 and S2 samples is 326 kA/cm^2^ and 317 kA/cm^2^, respectively. In the high magnetic field, the increase of *J*_C_ appears in the PeF of S2 samples, which is important for the future application of MgB_2_ bulks. It is clear that the PeF and PaF of S1 processed under lower prepared temperatures possess the optimum superconducting performance in a low magnetic field; however, *J*_C_ of the PeF of the S2 sample processed under higher prepared temperatures is more advantageous in a high magnetic field, which indicates that the preparation temperatures (950 °C and 975 °C) of the samples are appropriate. According to the report [5], the superconductor bulks MgB_2_ prepared at a temperature of 850 °C with the ex situ method using the SPS technique show the best *J*_C_ in the whole magnetic field compared with the bulks prepared at different temperatures. The value of *J*_C_ increases first and decreases then with the increase in the preparation temperature to 800 °C, 850 °C, 900 °C and 1000 °C, which further suggests that a suitable preparation temperature may exhibit a better superconducting property. Thus, it cannot be fully proved that a higher preparation temperature is harmful to the superconducting properties of MgB_2_ bulk in our work; further study, with a wider preparation temperature range, is required.

**Figure 5:**
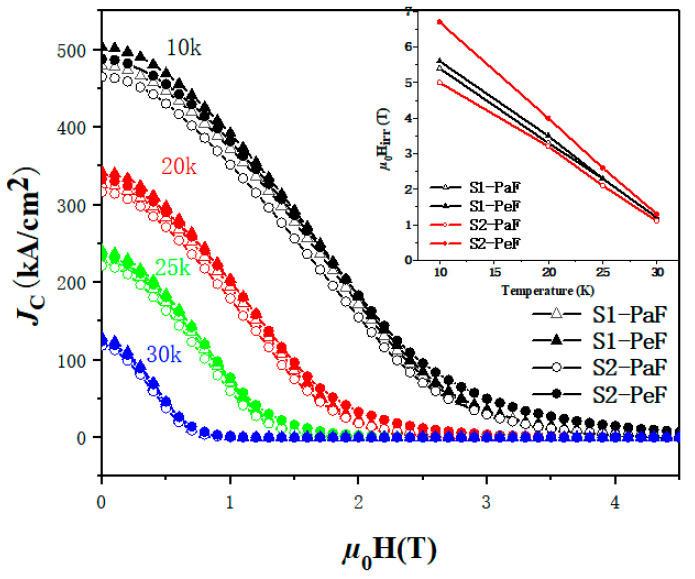
The *J*_C_-*μ*_0_H curves of the magnetic field and critical current density at different temperatures (10 K, 20 K, 25 K, and 30 K) of two surface PeF and PaF, of SPSed MgB_2_ bulk samples. The inset shows the values of *J*_C_ in an irreversible field under different temperatures.

*J*_C_ of the two samples at the different processed temperatures as a function of the applied field is given in Figure 5. In addition, the embedded diagram shows their irreversible field, represented by *μ*_0_*H*_irr_, which is defined as the field when *J*_C_ reaches 100 A/cm^2^, under different processed temperatures. It is clear that the *J*_C_ of the S1-PeF possesses the highest *J*_C_ in a low magnetic field, and the S2-PeF enhances the *J*_C_ in a high magnetic field, accompanied by the highest *μ*_0_*H*_irr_ at 20 K. The significant enhancement of *J*_C_ at a temperature of 10 K appears in a high magnetic field, which will be beneficial to future applications of MgB_2_ bulks.

### 3.2. The Microstructure and Crystal Size of MgB_2_ Samples at Different Preparation Temperatures (950 °C and 975 °C)

**Figure 6:**
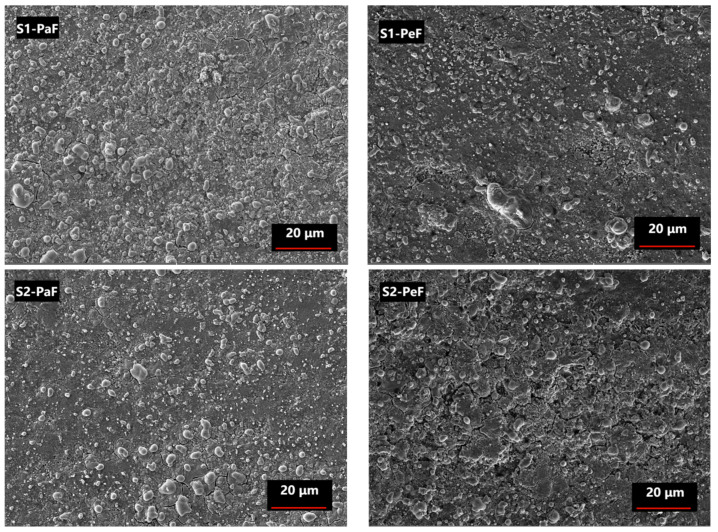
SEM diagrams from the PaF (**left**) and the PeF (**right**) of the SPSed MgB_2_ bulks prepared under 50 MPa pressure at 950 °C (S1) and 975 °C (S2), respectively.

Figure 6 shows a SEM diagram of the PaF (left) and PeF (right) of the SPSed samples. It is found that some large and white round and oval particles appear in the PaF of both samples, which are oxidative metamorphic MgB_2_ grains or MgO secondary particles [7,8]. It has been shown that the size of the effective pinning centers is similar to the coherence length of normal MgB_2_ (about 12 nm) [31], which results in the improvement of superconducting properties. Most oxidative metamorphic MgB_2_ grains and MgO secondary particles are too large to be effective pinning centers, which are harmful to the connectivity between normal MgB_2_ grains and have a negative effect on *J*_C_, as shown in Figure 4, with the decrease of *J*_C_ in the PaF of both samples. There are more grain boundaries in the PeF of both samples, which are prone to GBP to improve the superconducting properties of MgB_2_ bulks [28,29], which is consistent with the higher *J*_C_ of the PeF in both samples, as shown in Figure 4 and Figure 5. Moreover, the existence of more small-sized particles in the PeF of the S1 sample may be related to the highest self-field *J*_C_, as shown in Figure 4.

**Figure 7:**
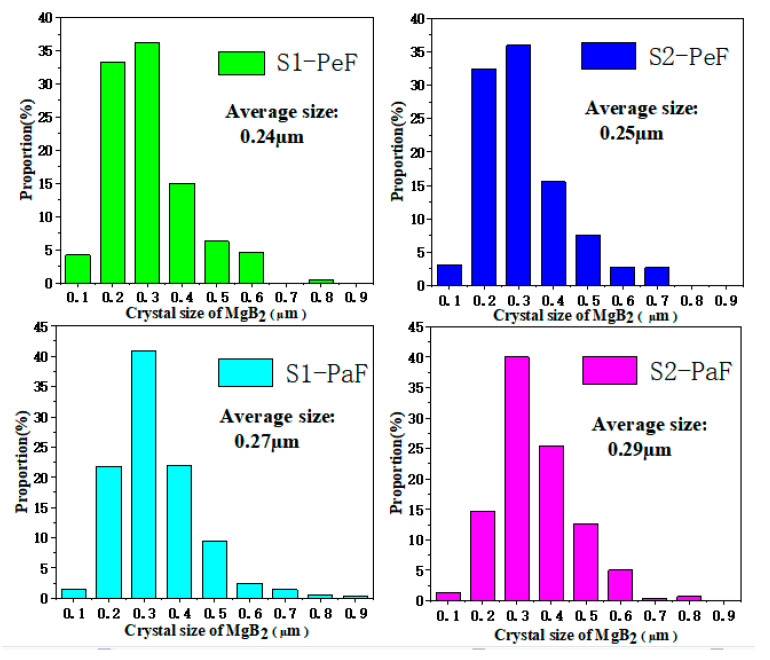
The crystal size distribution and average size of SPSed MgB_2_ bulks measured by Nano Measurer 1.2 software.

Figure 7 shows the statistical results of the MgB_2_ crystal size in the SEM pictures (Figure 6) based on Nano Measurer 1.2 software. The average sizes of samples are listed in Table 1. The horizontal axis is the range of the crystallite size, and the vertical axis is the percentage of the number of MgB_2_ grains in a certain size range based on the total statistical MgB_2_ grains in each specimen. It is easy to see that S1-PeF has the smallest average size (0.24 µm), and S2-PaF has the largest average size (0.29 µm). A smaller crystal size means more MgB_2_ particles and grain boundaries per unit volume, which can increase the number of effective pinning centers and further improve the *J*_C_ of MgB_2_ [32]. This is consistent with the trend of *J*_C_ in Figure 3 and Figure 4, which confirms that the *J*_C_ is greatly influenced by the crystal size of the SPSed MgB_2_ bulks.

### 3.3. The Flux-Pinning Mechanism

**Figure 8:**
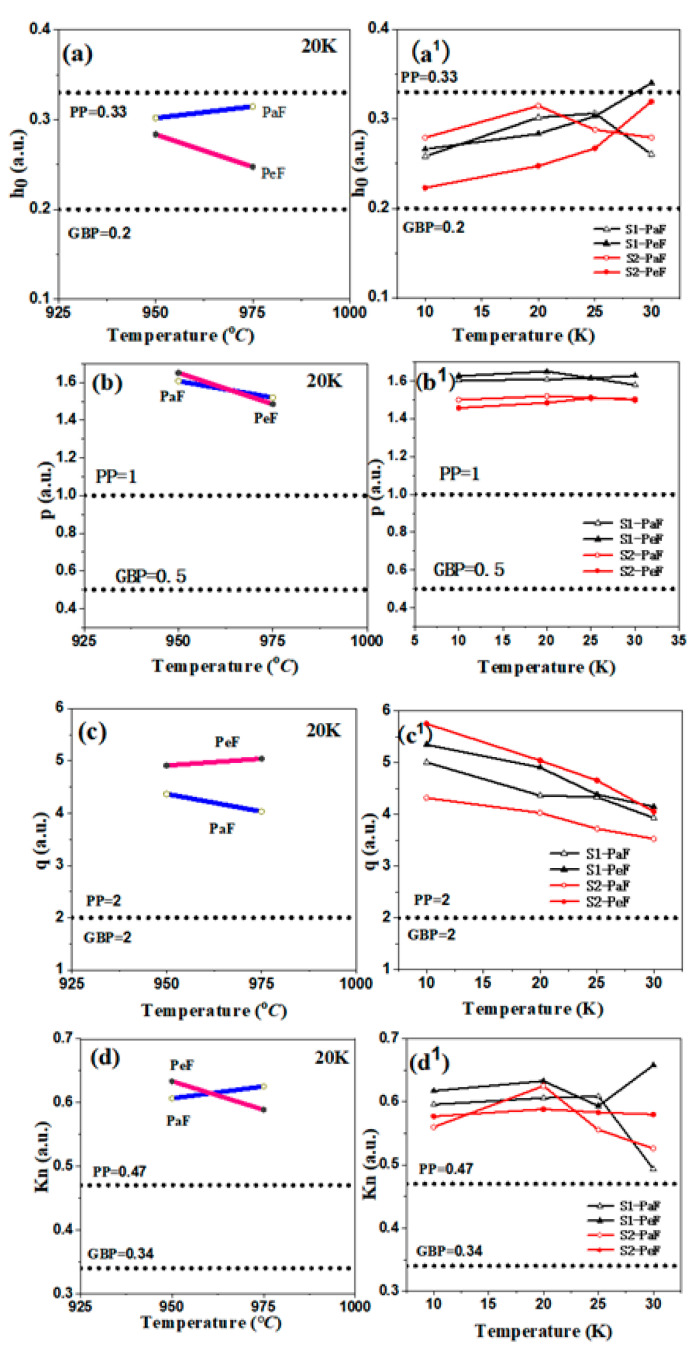
The plots of the parameters related to the pinning force: *h*_0_ (**a**), *p* (**b**), *q* (**c**), and *K*_n_ (**d**) as functions of preparation temperature at 20 K; *h*_0_ (**a^1^**), *p* (**b^1^**), *q* (**c^1^**), and *K*_n_ (**d^1^**) as functions of test temperature. The theoretical values of grain boundary pinning (GBP) and point pinning (PP) for reference are represented by dotted lines.

To study the flux-pinning mechanism, two scaling procedures should be used to identify the dominant pinning mechanism from the peak position [33,34]. According to Dew-Hughes [33], one of them is the universal law *F*_p_ = *Ah^p^*(1 − *h*)*^q^* (where A is constant, and *F*_p_ is the volume pinning force. Another equation is *F*_p_ = *μ*_0_H × *J*_C_ and h is a reduced field; it can be presented by the equation *h* = H/*H*_irr_). The law was chosen to fit the data of *F*_p_ and H/*H*_irr_. The parameter *h*_0_ is the field when *F*_p_ reaches its maximum, and the exponents *p* and *q* are fitted from the data of *F*_p_ and H/*H*_irr_, which analyzes the flux-pinning behavior more deeply and easily. Figure 8 shows the curves of the parameters related to the pinning force: *h*_0_ (a), *p* (b), and *q* (c) as a function of the preparation temperature and *h*_0_ (a_1_), *p* (b_1_), and *q* (c_1_) as a function of test temperature. The theoretical values of the GBP and PP for reference are represented by the dotted lines. For isotropic materials, GBP is identified by *h*_0_ = 0.2, *p* = 0.5, and *q* = 2, while PP is identified by *h*_0_ = 0.33, *p* = 1, and *q* = 2. However, this scaling procedure has its limitation for MgB_2_ materials, because it does not apply to untextured, anisotropic materials. Therefore, another scaling procedure, whose parameter *K*_n_ is presented by *K*_n_ = *h*_0_/*h*_n_ (where *h*_n_ refers to the field when *F*_p_ is halved), was proposed by Eisterer [34], to analyze the flux-pinning mechanism to achieve a more accurate flux-pinning mechanism behavior. As a modified scaling procedure, it can greatly reduce the influence of previously unknown parameters of anisotropy. The *K*_n_ values of 0.34 and 0.47 are defined for GBP and PP, respectively, and the plots of *K*_n_ (d) and (d_1_) as functions of the preparation temperature and the test temperature are shown in Figure 8. These two procedures mentioned above will be combined to discuss the pinning mechanism of the samples.

**Figure 9:**
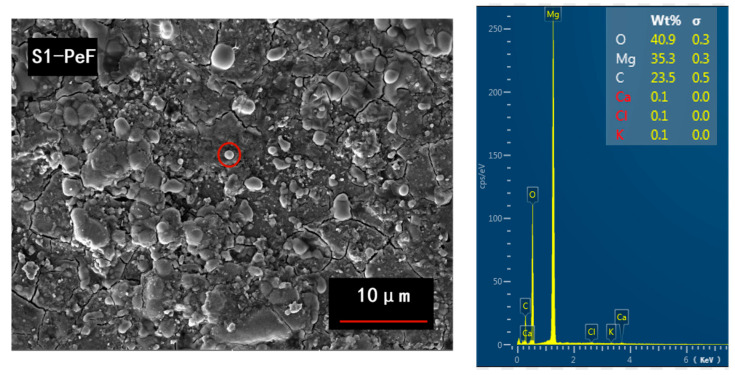
The EDS spectra (**right**) of the area circled in red (**left**).

It is obvious from Figure 8a,d that the values of the pinning force related to parameters *h*_0_ and *K*_n_ of the PeF are lower than those of the PaF, except for *K*_n_ of the PeF of S1, which means that the PeF has a stronger GBP than the PaF. Based on the previous report [7,35], the smaller crystallite size causes more grain boundaries per unit volume, which means a stronger GBP, as shown in the microstructure of Figure 6 and the smaller crystallite size of Figure 7, accompanied by a better superconducting performance of the PeF in both samples. The values of the pinning force-related parameters *h*_0_ and *K*_n_ of the PeF of S2 decrease with the increase in the preparation temperature of the MgB_2_ bulk samples, which means that S2 has a stronger GBP than S1. We can speculate that the crystallite size of S2 is smaller than the crystallite size of S1. In addition, according to this deduction, *J*_C_ of S2 should be higher than that of S1 in a magnetic field, but the trend of *J*_C_ is opposite to this rule, as shown in Figure 4 and Figure 5. This represents that GBP is not the only factor affecting *J*_C_ in the PeF of S1. There must be other factors that influence it—maybe the secondary phase particles in the suitable size, such as MgO or MgB_4_, etc. The right picture shows the chemical composition of the area circled in red in the left picture, as shown in Figure 9. From the EDS spectra, we can calculate the chemical formula and then verify the existence of MgO particles, which is confirmed by the XRD analysis of Figure 2. MgO particles can be effective pinning centers. In addition, both PP and GBP work on *J*_C_ at the same time to result in the improvement of *J*_C_ in the PeF of the S1 sample under the lower preparation temperature, as shown in Figure 4 and Figure 5. The discrepancy of S2 under higher preparation temperature in the parameters *h*_0_ and *K*_n_ is higher than that of S1, which is related to the increasing anisotropy of the SPSed samples, which accounts for the difference in properties between the PeF and PaF of S2. This is not conducive to practical application.

The relationship between the pinning mechanism and the critical current of MgB_2_ bulks has been studied, and further investigation of the relationship between pinning force-related parameters and *J*_C_ in high magnetic fields has been studied as well. It is inferred that more grain boundaries can enhance the *H*_c2_ [33,36], which can be presented by *H*_irr_ [30,36], so we can predict that stronger GBP can lead to higher *H*_irr_ and then improve *J*_C_ in high magnetic fields [7]. This coincides with the result, as shown in Figure 4 and Figure 5, which has the best performance in a high magnetic field in *J*_C_ of S2-PeF.

It is evident that the values of *h*_0_ and *K*_n_ in the PeF of both samples, as shown in Figure 8a^1^, become larger with the increase in the test temperature, from 10 K to 30 K, which means the PP grows stronger. The stronger PP indicates that much more effective pinning centers exist, and these effective pinning centers can bring higher *J*_C_. This is consistent with our observation of the MgO second phases in Figure 2, which can be effective pinning centers. This theory is consistent with the performance of *J*_C_, as shown in Figure 5. We can notice that the values of *h*_0_ and *K*_n_ of the PaF in both samples from Figure 8a^1^,d^1^ are not a monotonous trend, but rather a fluctuation, which is not consistent with the conclusion that PP as the main pinning becomes stronger as the test temperature increases, which means that the values of *h*_0_ and *K*_n_ show an overall increasing trend with increasing test temperature [19,36,37]. Maybe some evidence can be found from some studies with small temperature intervals [21,37]. The change in anisotropy or percolation with temperature may cause this temperature dependence of the pinning mechanism [7].

## 4. Conclusions

In this study, we prepared two MgB_2_ bulk samples, with a disc shape of 20 mm in diameter and 3 mm in thickness, at different temperatures of 950 °C (marked S1) and 975 °C (marked S2) for 2 h under 50 MPa pressure using the spark plasma sintering technique. The properties of the samples were analyzed according to the results of magnetization measurement and SEM diagrams. Critical temperature (*T*_C_), critical current density (*J*_C_), microstructure, and the pinning mechanism of the perpendicular facets (PeF) and parallel facets (PaF) to the compression direction of MgB_2_ bulk samples are all included. The onset of critical temperature is around 37.5 K and the transition width is about 1 K, which indicates all samples exhibit good crystallinity and homogeneity. In terms of *T*_C_, the onset transition temperature, *T*_c,onset_, of S1 is slightly higher than that of the S2 sample, which may be related to the good performance of the S1 sample. In terms of *J*_C_, the critical current density of all MgB_2_ bulk samples decreases with the increase of the applied field. In a low magnetic field, S1-PeF has the highest *J*_C_ (342 kA/cm^2^), followed by S2-PeF (333 kA/cm^2^), S1-PaF (326 kA/cm^2^), and S2-PaF (317 kA/cm^2^) in self-field at 20 K, respectively. The crystal size of the samples is 0.24 µm (S1-PeF), 0.25 µm (S2-PeF), 0.27 µm (S1-PaF), and 0.29 µm (S2-PaF), respectively. Thus, the values of *J*_C_ and the crystal size of samples obey the rule that a smaller crystal size can improve the *J*_C_ of MgB_2_ samples. However, in a high magnetic field, the S2-PeF has the highest *J*_C_, which contradicts the rule above. It illustrates that other factors can affect *J*_C_. According to the pinning mechanism, we find the factor is GBP, where stronger GBP can improve the *J*_C_ in a high magnetic field. In terms of the pinning mechanism, according to the analysis of the trend of the parameters *h*_0_, *p*, *q*, and *K*_n_, the PeF has a stronger GBP than the PaF, which is related to the smaller crystallite size, resulting in more grain boundaries per unit volume. Therefore, PeF shows better superconducting performance with an increase in the preparation temperature. The existence of a slightly higher discrepancy of properties occurs in the S2 sample. In addition, with the increase in temperatures, PP grows stronger, bringing higher *J*_C_. It is clear that the PeF and PaF of S1 processed under a lower prepared temperature possess the optimum superconducting performance in a low magnetic field; however, *J*_C_ of the PeF of the S2 sample processed under a higher prepared temperature is more advantageous in a high magnetic field. Therefore, further suitable preparation temperatures to fabricate MgB_2_ superconductor bulks using the SPS technique will appear in future experiments.

## Figures and Tables

**Table 1:** The lattice parameters and average grain sizes of two crystal facets (PaF and PeF) of samples prepared using SPS technology at 950 °C (S1) and 975 °C (S2) for 2 h under 50 MPa pressure.

Sample	Lattice Parameters (Å)	Average Particle Size (µm)
	a/c	PaF/PeF
S1 (950 °C)	3.0847/3.5232	0.27/0.24
S2 (975 °C)	3.0811/3.5223	0.29/0.25

## Data Availability

The data presented in this study are available on request from the corresponding author.
